# Association between patterns of sedentary time and academic performance in adolescents: the mediating role of self-concept

**DOI:** 10.1590/1984-0462/2022/40/2021106IN

**Published:** 2022-05-06

**Authors:** Maria Raquel de Oliveira Bueno, André de Oliveira Werneck, Danilo Rodrigues Pereira da Silva, Adewale Luqman. Oyeyemi, Lidyane Ferreira Zambrin, Romulo Araújo Fernandes, Helio Serassuelo, Marcelo Romanzini, Enio Ricardo Vaz Ronque

**Affiliations:** a Universidade Estadual de Londrina, Londrina, PR, Brazil.; b Universidade de São Paulo, São Paulo, SP, Brazil.; c Universidade Federal de Sergipe, São Cristóvão, SE, Brazil.; d University of Maiduguri, Borno State, Nigeria.; e Universidade Estadual Paulista “Júlio de Mesquita Filho”, Presidente Prudente, SP, Brazil.

**Keywords:** Sedentary behavior, Self concept, Academic performance, Adolescent, Comportamento sedentário, Autoestima, Desempenho acadêmico, Adolescente

## Abstract

**Objective:**

To analyze the association between the pattern of sedentary time (bouts and breaks) with academic performance, with an emphasis on the mediating role of self-concept.

**Methods:**

Participants in the cross-sectional study were 394 adolescents (208 girls), aged 10–14 years, from sixth grade from Londrina, Paraná, Brazil. The sedentary time pattern was measured through accelerometry. Definitions: sedentary bouts — uninterrupted periods of sedentary behavior; breaks — non-sedentary period between two sedentary bouts. The self-concept was estimated using the Piers-Harris II Inventory. Academic performance was obtained by school grades.

**Results:**

Short sedentary bouts were associated with higher academic performance in boys (1–4 minutes: β=0.035, p=0.007) and girls (1–4 minutes: β=0.031, p=0.014; 5–14 minutes: β=0.054, p=0.001). Long bouts (30 minutes) were associated with lower academic performance in boys (β=-0.023; p=0.011) and girls (β=-0.032; p<0.001). For girls, total and intellectual self-concept mediated the association between all sedentary pattern and academic performance (bouts 1–4 minutes [total: 39% and intellectual: 42.8%]; bouts 5–14 minutes [total: 21.5% and intellectual: 35.4%]; bouts ≥30 minutes [total: 22.6% and intellectual: 32.3%]; and breaks [total: 38.9% and intellectual: 40.7%]). For boys, the total (56.4%) and intellectual (82.9%) self-concept mediated only the association between bouts of 5–14 minute and academic performance.

**Conclusions:**

The pattern of sedentary time is associated with academic performance in adolescents and this association is mediated by self-concept, especially in girls.

## Resumo

**Objetivo:**

Analisar a associação entre o padrão do tempo sedentário (*bouts e breaks*) e o desempenho acadêmico, com ênfase no papel mediador do autoconceito.

**Métodos:**

Participaram deste estudo transversal 394 adolescentes (208 moças), de 10 a 14 anos, da sexta série de Londrina, Paraná, Brasil. O padrão do tempo sedentário foi obtido por meio de acelerometria. Definições: bouts sedentários — períodos ininterruptos em comportamento sedentário; breaks — período não sedentário entre duas séries sedentárias. O autoconceito foi estimado usando o Inventário Piers-Harris II. O desempenho acadêmico foi obtido pelas notas escolares.

**Resultados:**

Bouts sedentários curtos foram associados com maior desempenho acadêmico em rapazes (1–4 minutos: β=0,035, p=0,007) e moças (1–4 minutos: β=0,031, p=0,014; 5–14 minutos: β=0,054, p=0,001). Bouts longos (30 minutos) foram associados a menor desempenho acadêmico em rapazes (β=-0,023; p=0,011) e moças (β=-0,032; p<0,001). Para as moças, o autoconceito total e intelectual mediou a associação entre todos os padrões sedentários e o desempenho acadêmico (bouts de 1–4 minutos [total: 39% e intelectual: 42,8%]; bouts de 5–14 minutos [total: 21,5% e intelectual: 35,4%]; bouts ≥30 minutos [total: 22,6% e intelectual: 32,3%]; e breaks [total: 38,9% e intelectual: 40,7%]). Para os rapazes, o autoconceito total (56,4%) e intelectual (82,9%) mediaram apenas a associação entre bouts de 5–14 minutos e desempenho acadêmico.

**Conclusões:**

O padrão do tempo sedentário está associado ao desempenho acadêmico em adolescentes, e essa associação é mediada pelo autoconceito, principalmente em moças.

## INTRODUCTION

Besides the documented evidence on the association between physical inactivity and several negative health outcomes,^1^ there is emerging evidence on the harmful effects of sedentary behavior as a distinct dimension of human behavior.^2^ Even during adolescence, sedentary behavior has been associated with increased overweight and obesity, metabolic and cardiovascular markers,^1^ declined cognitive function,^3^ depressive symptoms, and reduced self-esteem and self-concept.^4^

In addition to these deleterious health outcomes, sedentary behavior has also been associated with academic performance^5^ in children and youth. However, previous studies are inconsistent on the direction of influence of sedentary behavior on academic performance. Total device-measured sedentary time (ST) was positively associated with academic performance^6,7^ while no association was observed in other studies.^8,9^ These inconsistencies could partly be explained by different measures of academic performance used in the studies, as well as by the fact that the association between ST and academic performance is domain/pattern dependent.^8^

More so, different patterns of ST in terms of bouts (uninterrupted amount of time during the behavior) and breaks (interruption of the behavior with at least light physical activity) may impact the academic achievement among children.^10^ However, the association between durations of bouts/amounts of breaks of ST in free-living conditions and academic achievement is unclear and has not been explored among adolescents.

In addition, the relationship between ST and academic performance is complex and may occur through multiple pathways. In order to inform effective intervention, it is important to understand the potential psychosocial mediators of this relationship. Self-concept — defined as the perception of a person about him/herself —^11^ was associated with both sedentary behavior^12^ and academic performance,^13^ suggesting it could represent an important mediator of the association between ST patterns and academic performance. The self-concept has been directly related to academic achievement, with a mutual reinforcement between them.^13^ However, this potential pathway has not been tested.

Thus, the aim of this study was to verify the association between ST patterns (bouts and breaks) and academic performance among adolescents aged 10 to 14 years, with emphasis on the mediating role of self-concept on this association.

## METHOD

This was a school-based cross-sectional study, involving adolescents (both sexes) aged between 10 and 14years old, in the sixth grade of elementary school in public schools in Londrina, Paraná, Brazil. Details of the sample selection process are described in the study of Bueno et al.^14^ The exclusion criteria were: students who had any physical limitations, or who were being treated for any disease or injury during the study, or who refused to use the accelerometer; aged over 15 years. In addition, the measures were replicated in a sub-sample (n=25), with an interval of one week, to estimate the agreement of the instruments (through *Kappa* coefficients [k] and intraclass correlation coefficient [ICC]). Both the students and their guardians signed the informed consent to participate. The local Ethics Committee approved all the procedures of this study according to Declaration of Helsinki (process number 1.281.324, October 2015).

Academic performance was assessed through semester grades of the following school subjects: Brazilian Portuguese, Mathematics, Science, History and Geography. The grades refer to numerical assessment on a scale of 0–10, in which grades below 6 denote the student’s failure and 10 denotes excellent knowledge and skills. Based on the information of the students’ performance, the averages of the disciplines were calculated, and an mean of the five school subjects was adopted as indicator for the analyses. The academic performance was provided by the education services of the city of Londrina.

ActiGraph (ActiGraph, Pensacola, FL, USA) GT3X and GT3X-Plus models were used to estimate ST patterns. Participants were instructed to wear the device on the right side of the waist (near the iliac crest) via an elastic belt for seven consecutive days and only remove it during shower, aquatic activities and sleeping time. The accelerometers were configured to collect information in one-second epochs (model GT3X) or at 30 Hz (model GT3X-Plus). After the monitoring period, the equipment was collected and the data stored in the ActiLife computer program (version 6.8.2). Subjects who had at least four complete days of data (>480 min/day, with at least one weekend day) registered by the accelerometer were included in the analyses. The criterion of 60 min of consecutive zeros was utilized to determine the non-wear time.^15^

ST was classified using cut-points developed for ActiGraph vector magnitude counts (180 counts.15s^-1^) in Brazilian adolescents.^16^ Bouts were defined as uninterrupted periods in sedentary behavior (drop time=0) with durations of 1–4 minutes, 5–14 minutes and ≥30 minutes. Breaks were defined as the non-sedentary period between two sedentary series.^17^ For analytic purposes, total ST and time accumulated in bouts were expressed as percentage values (percentage of total time using accelerometer), while breaks were expressed as mean frequency by hour of ST (breaks.hour^-1^).

Self-concept was evaluated through the Piers-Harris II inventory, adapted to the Brazilian Portuguese language by Serassuelo Junior.^18^ This instrument can be used with samples ranging from seven to eighteen years and consists of an inventory with 60 affirmations, with alternative answers (yes or no). The instrument provides, in addition to the total self-concept, six domain scales: behavior, intellectual and school status, physical appearance and personal attributes, anxiety free, popularity, happiness and satisfaction. Participants answered the complete inventory; however, for the present study, the following domains were used: total self-concept (TOT), which reflects the general measure of self-concept, with high results indicating a favorable measure of self-esteem and low results indicating low self-esteem; intellectual and school status (INT), which reflects how children evaluate their skills in relation to intellectual and academic tasks, as well as their satisfaction with the school and their expectations about future achievements; and anxiety-free (FRE), which reflects and evaluates anxiety and behaviors such as worry, nervousness, shyness, sadness and fear.^19^ The procedures described in the inventory manual were followed.^19^ The self-concept scales presented high agreement (k=0.81–1.0).^18^

The body mass was measured on a portable scale of the brand Seca^®^, model 813, with precision of 0.1kg, while the stature was determined in a Harpenden Holtain Limited^®^ portable stadiometer with a precision of 0.1cm, according to standardized procedures.^20^ Based on this information, body mass index was calculated. Socioeconomic status (SES) was estimated using the Brazilian Association of Research Companies questionnaire,^21^ which is composed by questions regarding the purchasing power, with rating ranging from 1 (low SES) to 6 (high SES). The moderate-to-vigorous physical activity (MVPA) was used as a covariate and classified using cut-points recorded by the vector magnitude of the accelerometer (MVPA≥757 counts.15s^-1^).^16^

The mentally-passive and mentally-active sedentary behavior was used as a covariate, and were assessed through questions about different sedentary behavior, which were asked through the generic questions: “Considering a typical weekday (Monday to Friday), how much time do you spend… (e.g. watch TV)?” and “Considering a typical weekend day (Saturday and Sunday), how much time do you spend… (e.g. watch TV)?”. These questions were asked for watching TV (ICC=0.90), watching DVD (ICC=0.33), using computer for leisure (ICC= 0.72), playing electronic games (ICC=0.54), studying (ICC=0.87) and reading (ICC=0.79), with six possible answers: (a) none, (b) less than 1 hour, (c) between 1 and 2 hours, (d) between 2.01 and 3 hours, (e) between 3.01 and 4 hours, (f) more than 4 hours. Mean time spent in each behavior was computed (e.g. less than 1 hour was transformed to 0.5 hour), and behaviors were divided into mentally-passive (watching TV, watching DVD, using computer for leisure) and mentally-active (playing electronic games, studying and reading) according to the expected cognitive demand. They were summed^22^ and two continuous indicators were created.

Descriptive statistics, with means as well as their respective standard deviations, were used to describe the sample, and differences between sexes were assessed by Mann-Whitney U test. Pearson correlation (separately by sex) was performed to explore the relationship between different self-concept, bouts and breaks of ST. Multiple linear regression was used to test the association between different ST patterns and academic performance regardless of potential confounders. Mediation analysis was conducted to assess the influence of self-concept and efficacy mediators in the association between different sedentary patterns and academic performance. Only the ST patterns that were significant predictors of academic performance were included in the mediation models. The Karlson Holm Breen method^23^ was used for the mediation. This method was applied in linear regression models and decomposes the total effect of a variable into direct and indirect effects. This estimation also provides the percentage of explanation by the potential influential factor (mediated percentage). All analyses were conducted in STATA 15.1, adopting p<0.05.

## RESULTS

From 680 eligible participants, 286 did not provide valid accelerometer data (failed to provide minimal data), totalizing a final sample of 394 adolescents. Despite the high level of missing data for device-measured ST, there were no substantial differences between all participants and the final sample for the main variables of the study. The final sample was composed of 394 adolescents (186 boys), with a mean chronological age of 11.9±0.7 years. Characteristics of sample according to sex are presented in Table 1. Girls presented higher academic performance (p=0.001) and lower moderate-to-vigorous physical activity (p<0.001) compared to boys.

**Table 1.** Characteristics of the sample.

	Boys (n=186)	Girls (n=208)	p-value
Chronological age (years)	11.9±0.7	11.8±0.6	0.418
Stature (cm)	150.8±8.7	152.6±7.4	0.006
Body mass (kg)	45.8±12.2	47.3±12.5	0.219
Body mass index (kg/m^2^)	19.1±4.2	20.1±4.3	0.596
Socioeconomic status (score)	4.5±1.2	4.4±1.2	0.204
Academic performance (score)	7.4±1.3	7.8±1.1	0.001
Use of accelerometer (min/day)	874.4±187.2	841.2±173.7	0.001
Sedentary time (% of wear time)	69.3±8.7	70.2±7.4	0.421
Bouts (% of sedentary time)			
1–4 minutes	29.6±8.4	30.9±7.8	0.141
5–14 minutes	28.8±6.3	28.2±4.9	0.398
≥30 minutes	16.4±11.0	15.1±9.7	0.422
Number of breaks per hour	11.3±2.5	11.7±2.3	0.121
MVPA (min/d)	86.9±33.7	66.8±25.1	<0.001
Mentally-active SB, h/d	3.4±2.6	3.0±2.3	0.113
Mentally-passive SB, h/d	4.4±2.9	3.9±2.4	0.095
Total self-concept	49.5±7.1	47.8±7.1	0.028
Intellectual self-concept	48.5±8.4	48.4±8.1	0.794
Anxiety self-concept	50.0±6.8	46.3±7.1	<0.001

MVPA: moderate to vigorous physical activity; SB: sedentary behavior; min/d: minutes a day; h/d: hours per day.

The association between different ST patterns (bouts and breaks) and academic performance is presented in Table 2. Shorter bouts (1–4 minutes among boys, 1–4 minutes and 5–14 minutes among girls) of ST were associated with a higher academic performance. Similarly, a greater number of breaks on ST were associated with higher academic performance, while longer bouts (≥30 minutes) of ST were associated with a lower academic performance in both sexes.

**Table 2.** Association of different bouts and number of breaks in sedentary time with academic performance.

	r^2^ adjusted	ß	95%CI	p-value
Boys (n=186)				
Sedentary time	0.114	-0.036	-0.084 to 0.012	0.138
1–4 minutes	0.130	0.035	0.009–0.060	0.007
5–14 minutes	0.095	0.014	-0.016 to 0.046	0.351
≥30 minutes	0.126	-0.023	-0.040 to -0.005	0.011
Number of breaks per hour	0.129	0.116	0.031–0.202	0.008
Girls (n=208)				
Sedentary time	0.050	-0.023	-0.065 to 0.019	0.284
1–4 minutes	0.087	0.031	0.006–0.056	0.014
5–14 minutes	0.108	0.054	0.021–0.088	0.001
≥30 minutes	0.120	-0.032	-0.050 to -0.015	<0.001
Number of breaks per hour	0.086	0.103	0.020–0.187	0.015

Adjusted by chronological age, body mass index, socioeconomic status, moderate to vigorous physical activity, mentally active sedentary behavior and mentally-passive sedentary behavior. 95%CI: 95% confidence interval.

The correlation between different patterns of ST and domains of self-concept is showed in Table 3. The most consistent correlations were found among girls for intellectual and anxiety-free domains of self-concept, as well as for the total self-concept.

**Table 3.** Correlation of different self-concept, bouts and breaks of sedentary time.

Boys (n=186)	1	2	3	4	5	6	7	8
1. Bout 1–4 minutes	1							
2. Bout 5–14 minutes	0.185*	1						
3. Bout ≥30 minutes	-0.801**	-0.652**	1					
4. Breaks per hour	0.990**	0.246*	-0.846**	1				
5. Total self-concept	0.037	-0.208*	0.038	0.032	1			
6. Intellectual self-concept	0.037	-0.173*	0.048	0.029	0.082**	1		
7. Anxiety self-concept	-0.036	-0.163*	0.060	-0.032	0.680**	0.045**	1	
8. Academic performance	0.170*	0.113	-0.181*	0.166*	0.241*	0.030**	0.171*	1

Girls (n=208)								
1. Bout 1–4 minutes	1							
2. Bout 5–14 minutes	0.106	1						
3. Bout ≥30 minutes	-0.801**	-0.575**	1					
4. Breaks per hour	0.992**	0.146*	-0.831**	1				
5. Total self-concept	0.226*	0.188*	-0.223*	0.223*	1			
6. Intellectual self-concept	0.187*	0.201*	-0.214*	0.176*	0.815**	1		
7. Anxiety self-concept	0.201*	0.097	-0.159*	0.204*	0.777**	0.448**	1	
8. Academic performance	0.039	0.236*	-0.147*	0.036	0.283	0.056	0.221*	1

*p<0.05; **p<0.00.

Table 4 shows the mediation models by self-concept domains of the association between different ST patterns and academic performance. TOT, as well as intellectual self-concept, mediated the association between all types of ST patterns and academic performance among girls (bouts 1–4 minutes [TOT=39% and INT=42.8%]; bouts 5–14 minutes [TOT=21.5% and INT=35.4%]; bouts ≥30 minutes [TOT=22.6% and INT=32.3%]; and breaks [TOT=38.9% and INT=40.7%]). Anxiety-free self-concept mediated the association between short bouts (1–4 min [23.8%]) and breaks (24.4%) with academic performance among girls. For boys, TOT (56.4%) and INT (82.9%) self-concepts only mediated the association between bouts of 5–14 minutes and academic performance.

**Table 4.** Different self-concept as mediators of the association between different bouts and breaks of sedentary time by sex.

		Total effect	p-value	Direct effect	p-value	Indirect effect	p-value	%mediator
Bout 1–4 minutes								
Total self-concept	Boys	0.030	0.018	0.032	0.013	-0.001	0.597	NA
	Girls	0.031	0.012	0.019	0.137	0.012	0.009	39.0
Intellectual self-concept	Boys	0.030	0.016	0.030	0.016	-0.001	0.979	NA
	Girls	0.031	0.009	0.018	0.141	0.013	0.013	42.8
Anxiety self-concept	Boys	0.030	0.019	0.034	0.009	0.004	0.164	NA
	Girls	0.031	0.013	0.024	0.065	0.007	0.044	23.8
Bout 5–14 minutes								
Total self-concept	Boys	0.018	0.249	0.027	0.079	-0.010	0.040	56.40
	Girls	0.054	0.001	0.043	0.010	0.012	0.031	21.5
Intellectual self-concept	Boys	0.018	0.239	0.032	0.037	-0.015	0.012	82.9
	Girls	0.054	<0.001	0.035	0.028	0.019	0.006	35.4
Anxiety self-concept	Boys	0.018	0.256	0.022	0.166	-0.004	0.192	NA
	Girls	0.054	0.001	0.050	0.001	0.003	0.231	NA
Bout ≥30 minutes								
Total self-concept	Boys	-0.021	0.014	-0.024	0.007	0.002	0.247	NA
	Girls	-0.032	<0.001	-0.025	0.005	-0.007	0.017	22.6
Intellectual self-concept	Boys	-0.021	0.013	-0.024	0.004	0.035	0.183	NA
	Girls	-0.032	<0.001	-0.022	0.011	-0.010	0.006	32.3
Anxiety self-concept	Boys	-0.021	0.016	-0.023	0.008	0.002	0.212	NA
	Girls	-0.032	<0.001	-0.029	0.001	-0.004	0.094	NA
Number of breaks per hour								
Total self-concept	Boys	0.101	0.018	0.106	0.013	-0.005	0.542	NA
	Girls	0.103	0.013	0.063	0.141	0.040	0.010	38.9
Intellectual self-concept	Boys	0.101	0.016	0.103	0.014	-0.002	0.865	NA
	Girls	0.103	0.009	0.061	0.130	0.042	0.018	40.7
Anxiety self-concept	Boys	0.101	0.019	0.113	0.009	-0.012	0.172	NA
	Girls	0.103	0.015	0.078	0.071	0.025	0.043	24.4

Bouts are represented in % of sedentary time. Breaks are represented as n/h. bold values represents p<0.05. Adjusted by chronological age, body mass index, socioeconomic status and moderate to vigorous physical activity. %mediation was only estimated for significant indirect effect and total effects. NA: not applicable.

## DISCUSSION

The present study analyzed (a) the academic performance between ST patterns (bouts and breaks) and academic performance in adolescents, and (b) the mediating role of self-concept in this association. We found that higher time spent in short bouts and a greater number of breaks in ST were associated with higher academic achievement. On the other hand, bouts ≥30 min were associated with lower academic performance. In addition, the self-concept mediated the associations between ST patterns and academic performance in both sexes, but more consistently in girls.

Interestingly, while patterns of ST were associated with academic achievement, it was observed that total ST was not, which corroborates previous studies.^8,9^ This result reinforced current ST guideline that emphasized that ST patterns and not just the total amount may be important for health outcomes.^24^ Moreover, these results may be related to the fact that device measurement does not provide specific contexts of ST, and some sedentary activities, such as reading and homework, may benefit academic achievement. Even there was an association between patterns of ST and academic performance, the magnitude of the associations was generally low, with patterns of ST explaining between 7 and 17% of the variation in the academic performance. Although the proportion is low, it must be highlighted that there are several determinants of academic achievement (academic skills, teacher perception, quality and quantity of academic teaching, and family and school environment)^25^ and a variation between 7 and 17% can be considerable in this context.

It has been reported that adolescents accumulated longer bouts of ST especially outside the school period.^26^ This pattern of ST tends to be spent in mentally-passive behaviors as TV-viewing, which in turn is associated with lower academic performance^8,9^ and cognition,^27^ which would justify the inverse association between longer bouts of ST and academic performance in the present study. This can be explained by time displacement hypothesis that excessive screen time could replace activities involving learning opportunities such as reading books, doing homework, sleeping well, all of which can positively influence academic performance.^28^ Moreover, screen time was negatively correlated with functional connectivity in regions related to language, visual processing and cognitive control in adolescents.^27^ Despite that, in this study, the ST pattern was associated with academic performance independent of the mentally-passive and mentally-active ST.

Therefore, more ST breaks should be encouraged in this age group. Short bouts are closely associated with interruption of ST and consequent muscle contraction would result in increased blood flow, release of growth factors and myocytes, which could increase brain activation, improve brainstorming and memorization,^3^ and contribute to a better academic performance.

In addition to the potential biological pathways that could link ST patterns and academic achievement, in this study, the mediator role of self-concept was tested in this association. Although little is known about this, it is possible to speculate that longer ST bouts is associated with reduced self-concept and self-esteem^29^ and, consequently, poor academic performance. Most of the studies that investigated the associations between ST and self-concept have analyzed only the physical self-concept.^12^ However, intellectual self-concept has shown real importance in academic outcomes in adolescents,^30^ as it reflects the adolescents’ perception of their abilities in relation to intellectual and academic tasks, as well as their satisfaction with the school and the expectations they have about future achievements.^19^

Also, self-concept partially mediated the association between all types of ST patterns (shorter and longer bouts and breaks) and academic performance among girls and shorter bouts and academic performance among boys. This result indicates a specific path among sexes and that self-concept seems an important variable to consider in order to understand the association between lifestyle behaviors and academic outcomes. In addition, screen time has been associated with negative effects on mental health indicators, such as self-esteem, depressive symptoms, psychological well-being and physical self-concept.^4^ This negative association may be related to increased exposure to social media, social isolation, depression, and cyberbullying.^31^ Further studies are needed to clarify how self-concept can affect the academic achievement and other psychological mediators of this association.

Therefore, it was used the global average of five subjects as it includes a wider range of skills, as used in other studies.^6,9^ However, it may be that different disciplines have different relationships with sedentary behavior,^32^ which cannot be verified in the present study, since it was used a single global average of academic performance.

The findings in this study should be interpreted in the light of potential limitations. Firstly, due to the cross-sectional design, it was not possible to infer causality and consequence. In addition, although many studies use school grades as a measure of academic performance,^7–9^ it can be biased due to its relationship not only to academic skills but also to teacher perception, quality and quantity of academic teaching, family and school environment, and even cultural factors.^25^ The level of missing data for device-measured ST was also high; however, there were no substantial differences between the total and final samples. Finally, the analyses by other potential unmeasured confounders as self-esteem and mood were not adjusted. On the other hand, this study has some important strengths. It was adopted a device measurement of the ST (accelerometry), which allowed an analysis of both the total ST and the ST patterns and adjusted the analyses by mentally-passive and mentally-active sedentary behavior. Besides that, the participation of a representative sample of students allows us a robust generalization of our findings to the studied population.

In conclusion, longer bouts of ST were associated with poorer academic performance while short bouts and breaks were associated with better academic performance among adolescents. This association is mediated by self-concept, mainly in girls. Furthermore, the patterns of ST were associated with academic performance and total ST was not, confirming the importance of analyzing the ST pattern with academic variables and not just the total ST. Future longitudinal studies should be conducted to confirm prospective association between different ST bouts and academic performance in adolescence.
